# Secondary Syphilis as a Mimic of Cutaneous Small-Vessel Vasculitis: A Diagnostic Pitfall

**DOI:** 10.7759/cureus.102701

**Published:** 2026-01-31

**Authors:** Ahmer A Longi, Misbah Fazlani, Arsheena Mohamed, Zubair Edakkavil, Betsy Narayan

**Affiliations:** 1 Internal Medicine, Mediclinic Welcare Hospital, Dubai, ARE; 2 Internal Medicine, Mediclinic Welcare Hosptial, Dubai, ARE; 3 Rheumatology, Mediclinic Welcare Hospital, Dubai, ARE; 4 Dermatology, Mediclinic Welcare Hospital, Dubai, ARE

**Keywords:** cutaneous vasculitis, great mimicker, palm and sole rash, secondary syphilis, treponema pallidum

## Abstract

Secondary syphilis is renowned for its ability to mimic a wide range of dermatologic and systemic diseases, including vasculitides. Misinterpretation of its cutaneous manifestations can delay appropriate therapy and expose patients to unnecessary immunosuppression. We report the case of a young female who presented with an acute, maculopapular, and annular rash initially affecting the upper body, including the back, chest, and upper limbs, followed by involvement of the lower body. It was preceded by redness and inflammation of her eyes. Initial clinical evaluation supported a diagnosis of cutaneous small-vessel vasculitis, and systemic vasculitis was strongly suspected. However, an extended history revealed recent unprotected sexual contact a few weeks before the symptoms started. Serologic testing demonstrated positive non-treponemal and treponemal tests consistent with secondary syphilis. The patient was treated with intravenous ceftriaxone, resulting in rapid resolution of the rash and improvement of her symptoms, with an appropriate decline in non-treponemal titers on follow-up. This case illustrates how secondary syphilis can present with a vasculitis-appearing rash clinically and underscores the need to include syphilis in the diagnostic workup of small-vessel vasculitis. Routine sexual history-taking and early syphilis serology in adults with an unexplained vasculitis rash may prevent diagnostic delay, inappropriate immunosuppression, and ongoing transmission.

## Introduction

Syphilis, caused by the spirochete *Treponema pallidum* (*T. pallidum*), remains an important global health problem, with rising incidence in many regions despite the availability of effective antibiotic therapy. Recent surveillance data highlight the highest reported rates of syphilis in decades, emphasizing its continued clinical relevance and the need for heightened diagnostic vigilance [1]. Secondary syphilis typically develops weeks after primary infection and is often the first stage to come to clinical attention, given its systemic manifestations and characteristic mucocutaneous eruption (involving the skin and the moist linings inside the body, like the mouth, lips, eyes, or genitals). It has long been dubbed the “great imitator” because of its ability to mimic a wide range of dermatologic and systemic conditions.

The classic rash of secondary syphilis is described as a symmetric, non-pruritic, maculopapular or papulosquamous eruption (rash with small raised bumps and flaky/scaly skin) involving the trunk and frequently the palms and soles, often accompanied by generalized lymphadenopathy and mucosal lesions [2]. However, an expanding body of literature underscores the breadth of its clinical spectrum. A recent case report of a young HIV-positive man described painful target-shaped plaques confined to the genitalia as the presenting sign of secondary syphilis, illustrating how the disease may closely resemble erythema multiforme (red patches or “target/bullseye” spots) and other targetoid dermatoses [3]. In 2019, 21.5% of all syphilis infections were reported in heterosexuals, with almost equal distribution among heterosexual men and women [4].

From a clinician’s perspective, annular, targetoid, and purpuric lesions occupy a broad differential diagnosis that extends well beyond syphilis. Trayes and colleagues reviewed annular lesions in primary care and demonstrated that tinea corporis, erythema migrans, plaque psoriasis, erythema multiforme, lichen planus, nummular eczema, subacute cutaneous lupus erythematosus, granuloma annulare, immunoglobulin A (IgA) vasculitis, sarcoidosis, and secondary syphilis may all present with ring-shaped or targetoid plaques [5].

In this report, we describe a young female who presented with a maculopapular, annular rash, initially diagnosed as cutaneous small-vessel vasculitis. Subsequent serology established secondary syphilis with *T. pallidum*-associated leukocytoclastic vasculitis. We discuss this case in the context of the expanding literature on atypical cutaneous manifestations of secondary syphilis and propose a practical approach to integrating syphilis testing into the workup of suspected vasculitis.

## Case presentation

**Figure 1:**
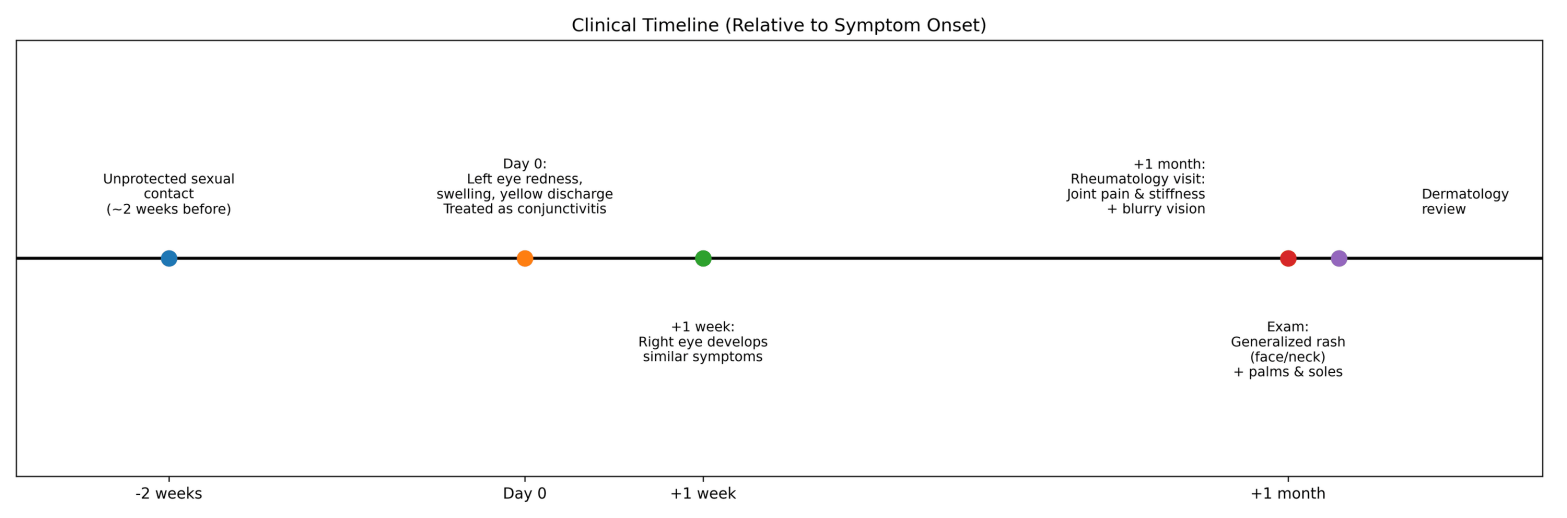
Clinical timeline of the case

A young lady in her early 20s, known to have iron deficiency anemia, presented to the clinic with complaints of feeling unwell. She had initially developed left eye symptoms with redness, swelling, and yellow discharge, which was treated as conjunctivitis. After one week, the right eye also showed similar symptoms. A month afterwards, she was seen in the rheumatology clinic for complaints of joint pain with stiffness preceded by skin rashes with ongoing visual blurriness. She had a generalized skin rash involving the face and neck, sparing neither the palms nor the soles. A dermatologist then reviewed her. She gave a history of having an unprotected sexual encounter with a partner two weeks before the symptoms started (Figure 1).

Physical examination

**Figure 2:**
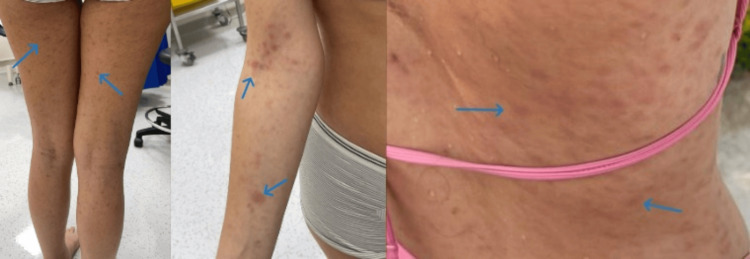
Erythematous maculopapular rash on the patient's lower limbs, right arm, and back

**Figure 3:**
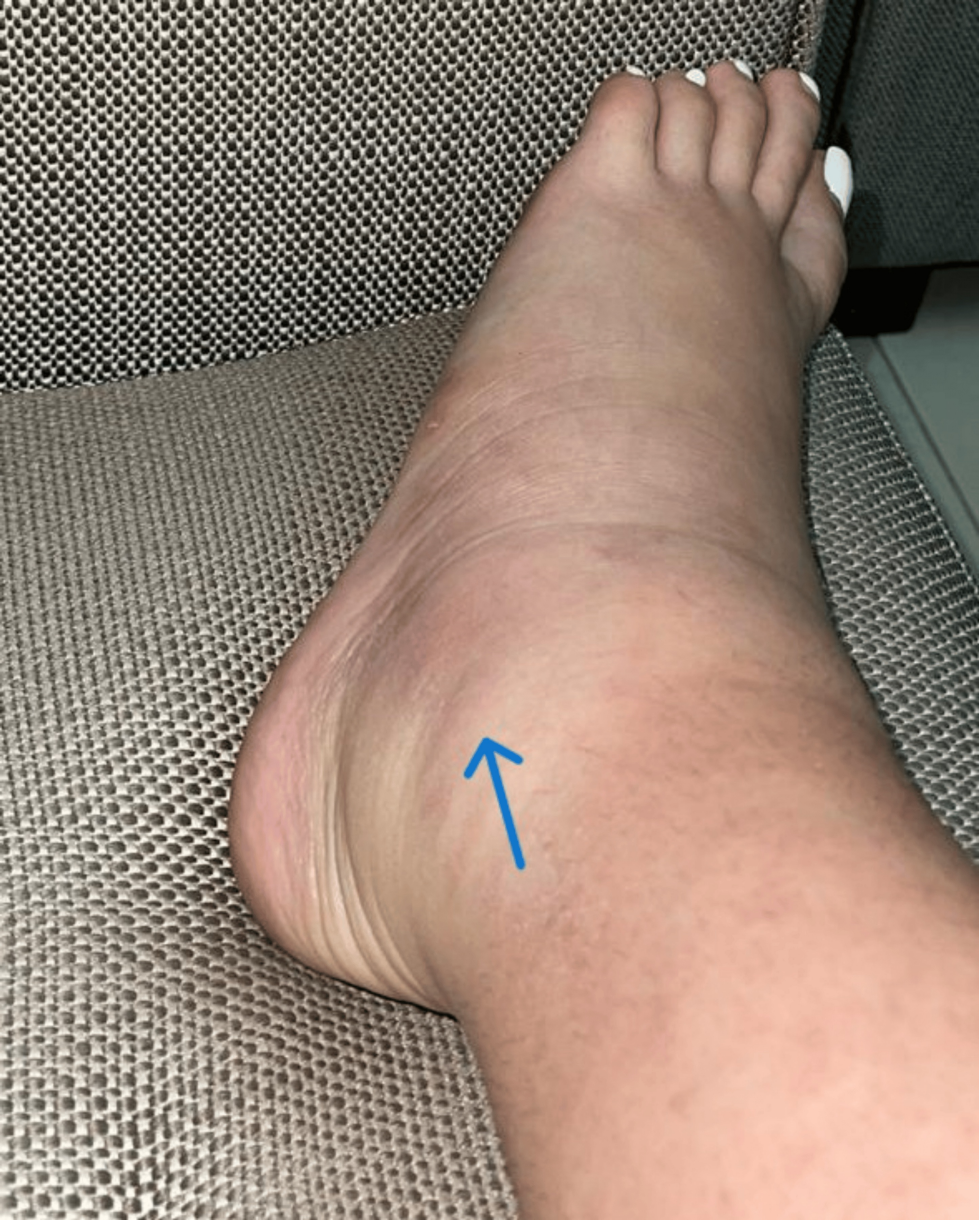
Right ankle synovitis

On initial examination, her vitals included blood pressure of 118/70 mmHg, pulse of 78 beats/min, respiratory rate of 16 breaths/min, and temperature of 37 °C. Her chest was clear to auscultation, and the abdomen was soft, non-tender, and non-distended, with her being alert and oriented to time, place, and person. She had a generalized skin rash, which appeared to be maculopapular with scaling involving the back, upper, and lower limbs. It was red, annular, and erythematous (Figure 2). The musculoskeletal exam showed her to have moderate bilateral ankle synovitis (Figure 3) along with mild bilateral wrist synovitis. On subsequent follow-up, her rash seemed to have increased and involved the face as well, along with photosensitivity.

Investigations

The patient's initial laboratory investigations were done (Table 1). Dermatology investigations showed positive results for syphilis with severe skin manifestations.

**Table 1:** Initial and follow-up lab investigations CRP: C-reactive protein; ESR: erythrocyte sedimentation rate; ALT: alanine aminotransferase; AST: aspartate aminotransferase; HCT: hematocrit; ANA: anti-nuclear antibody; HBsAg: hepatitis B surface antigen; HCV: hepatitis C antibody; HIV: human immunodeficiency virus; RPR: rapid plasma regain; TPHA: *Treponema pallidum* hemagglutination assay

Labs	First visit	Follow up	Units	Reference range
Hemoglobin	9.6	10.7	g/dL	11.5 – 16.0 g/dL
White cells	6.3	6.7	x10^9^/L	4.0 – 11.0 x10^9^/L
Platelets	292	322	x10^9^/L	150 – 450 x10^9^/L
CRP	43.1	54.1	mg/L	0.0 – 5.0 mg/L
ESR	40	NA/-	mm/hr	2 – 39 mm/hr
ALT	25.9	NA/-	U/L	< 35 U/L
AST	28.3	NA/-	U/L	< 35 U/L
HCT	28.8	NA/-	%	31-42%
ANA profile	Negative	NA/-	NA/-	Negative
HBsAg	NA/-	Non-reactive	NA/-	Non-reactive
HIV Ag/Ab 1-2	NA/-	Non-reactive	NA/-	Non-reactive
RPR	Reactive (1:32)	NA/-	NA/-	Non-reactive
TPHA	Positive (1:5120)	NA/-	NA/-	Negative
Treponemal antibody	Reactive	NA/-	NA/-	Non-reactive
ANA profile	Negative	NA/-	NA/-	Negative

Diagnosis and treatment

Based on the positivity of the syphilis diagnostic tests, the patient was prescribed intravenous ceftriaxone, to which she developed an allergic reaction (skin hives). She was treated with an antihistamine and a dose of steroids. She was also evaluated by ophthalmology (another facility), where she was also diagnosed with ocular syphilis. Meanwhile, she was advised to test for other sexually transmitted diseases, including human immunodeficiency virus (HIV), chlamydia, and gonorrhea. She was advised to continue on IV ceftriaxone, as her previous reaction was thought to be due to syphilis itself.

Follow-up

On follow-up after two weeks, she reported that the rash completely disappeared during the treatment. After two years, she again developed an annular rash on her lower abdomen. However, this time the rash was much more limited. Her laboratory tests done this time did not indicate that she had any active syphilis (Table 2).

**Table 2:** Laboratory investigations CRP: C-reactive protein; ALT: alanine aminotransferase; AST: aspartate aminotransferase; HCT: hematocrit; RPR: rapid plasma regain; TPHA: *Treponema pallidum* hemagglutination assay.

Labs	Results	Units	Reference range
Hemoglobin	12.5	g/dL	11.5 – 16.0 g/dL
White cells	6.6	x10^9^/L	4.0 – 11.0 x10^9^/L
Platelets	201	x10^9^/L	150 – 450 x10^9^/L
CRP	0.7	mg/L	0.0 – 5.0 mg/L
Creatinine	58.1	µmol/L	53 – 97 µmol/L
ALT	10.90	U/L	< 35 U/L
AST	19.70	U/L	< 35 U/L
HCT	36.8	%	31-42%
RPR	Non-reactive	NA/-	Non-reactive
TPHA	Positive (1:5120)	NA/-	Negative
Treponemal antibody	Reactive	NA/-	Non-reactive

## Discussion

Our patient’s presentation included palpable purpura and an annular rash on the whole body, accompanied by constitutional symptoms, which led appropriately to an initial working diagnosis of cutaneous small-vessel vasculitis with possible systemic involvement. In many settings, such a constellation would prompt an extensive evaluation for autoimmune, paraneoplastic, and drug-induced vasculitides and might lead rapidly to systemic corticosteroid therapy.

However, the differential diagnosis of palpable purpura and annular, targetoid lesions is broad, and infection-related etiologies are common and important. Trayes and colleagues highlighted that conditions such as tinea corporis, erythema multiforme, subacute cutaneous lupus erythematosus, IgA vasculitis, granuloma annulare, and secondary syphilis can all present with annular or targetoid plaques, some with superimposed purpura [5]. Without careful clinical correlation and appropriate laboratory testing, it would be difficult to distinguish between primary autoimmune vasculitis and vasculitis driven by infection or other triggers.

In our case, revisiting the history was crucial. The patient subsequently disclosed a recent unprotected sexual encounter several weeks before the onset of the rash. Basheer et al. emphasized that a limited or absent sexual history in patients with unusual rashes contributes significantly to missed or delayed diagnoses of syphilis, especially when the morphology overlaps with common dermatologic conditions such as tinea or eczema [4]. This underscores the importance of routinely including a non-judgmental sexual history in the assessment of adults with new, unexplained rashes or vasculitic eruptions.

Basheer et al. reported a heterosexual man with a pruritic papulosquamous rash initially diagnosed and treated as tinea, in whom secondary syphilis was ultimately confirmed, highlighting that pruritus, concurrent superficial fungal infection, and atypical distribution can all obscure the diagnosis [4]. In contrast, Sudibyo and Qurrohman described a 21-year-old woman with a more “textbook” presentation of diffuse, non-pruritic erythematous papules involving the palms, soles, and intertriginous regions, reinforcing the range from classical to highly atypical mucocutaneous disease even in young, otherwise healthy women [2].

Multiple reports now document syphilis masquerading as vasculitic disease at the bedside and under the microscope. Mohamed et al. described a 61-year-old man whose rapidly progressive purpuric eruption and renal involvement were initially attributed to leukocytoclastic vasculitis, but whose skin biopsy ultimately demonstrated a plasma cell-rich infiltrate with sparse neutrophilic nuclear dust and spirochetes highlighted by special stains, confirming secondary syphilis [6]. Rodriguez et al. reported rupioid (malignant) secondary syphilis in an HIV-positive patient, in whom hyperkeratotic, crusted plaques and histologic evidence of vasculitis complicated the picture and required close clinicopathologic correlation to reach the correct diagnosis [7]. Beyond the skin, Sangesland et al. published a case of neurosyphilis presenting with acute unilateral vision loss, scalp tenderness, elevated inflammatory markers, and a truncal maculopapular rash, initially managed as giant cell arteritis before syphilis serology and cerebrospinal fluid analysis clarified the diagnosis [8].

Basheer et al. highlighted how the absence of a sexual history and the assumption that a rash is purely dermatologic may contribute to delays in recognizing syphilis in non-traditional populations such as heterosexual men [4]. Given rising syphilis rates in diverse demographic groups, clinicians evaluating patients with vasculitic-appearing eruptions should be aware that syphilis can sit at the crossroads of infection, immune response, and vascular inflammation.

When syphilis serology was obtained in our patient, both non-treponemal and treponemal tests were strongly reactive, paralleling reports from other cases of secondary syphilis with atypical skin manifestations. Sudibyo and Qurrohman demonstrated that the combination of a reactive Venereal Disease Research Laboratory (VDRL) and a high-titer treponemal test (*Treponema pallidum* hemagglutination assay (TPHA)) provided definitive evidence of secondary syphilis in a young woman with diffuse mucocutaneous papules involving the palms, soles, and intertriginous regions [2]. In our case, a positive unprotected sexual history led to testing for syphilis. The interpretation of rapid plasma regain (RPR) and TPHA can be understood using the given information (Table 3).

**Table 3:** Interpretation of syphilis diagnostic tests RPR: rapid plasma reagin; TPHA: *Treponema pallidum *hemagglutination assay; -: negative; +: positive

Common result pattern	Most likely interpretation	Practical next step (typical)
RPR − / TPHA −	No serologic evidence of syphilis or very early infection	If recent exposure/symptoms: repeat in two to four weeks
RPR + / TPHA +	Syphilis is likely (active or previously treated)	Stage clinically; treat as indicated; use baseline RPR titer for follow-up
RPR + / TPHA −	Usually false-positive RPR; less often, very early syphilis	Repeat testing and/or confirm with another treponemal assay, depending on risk
RPR − / TPHA +	Past treated syphilis (common), late/latent syphilis, very early infection, or rarely false-positive treponemal test	Correlate with history; consider the second treponemal test and manage based on risk/history

This clinicopathologic correlation closely mirrors the experience reported by Mohamed et al., who described a 61-year-old man with rapidly progressive purpuric papules and acute kidney injury in whom leukocytoclastic vasculitis was initially favored. Spirochetal staining of the skin biopsy subsequently highlighted *T. pallidum* organisms, establishing secondary syphilis as the unifying diagnosis and altering management [6]. Similarly, Rodriguez et al. reported the case of a patient with rupioid (malignant) secondary syphilis in the setting of advanced HIV infection, whose hyperkeratotic plaques demonstrated vasculitic changes histologically. In that case, communication between clinicians and the dermatopathologist, combined with serologic testing, was necessary to recognize syphilis as the cause of a highly atypical vasculitic eruption [7].

The range of cutaneous morphologies that secondary syphilis can adopt is further illustrated by reports of targetoid, granulomatous, and papulosquamous patterns. Puttur et al. described a young man with HIV infection and concurrent molluscum contagiosum whose secondary syphilis manifested as painful target-shaped plaques on the genitalia, a striking departure from the typical non-tender, diffuse rash [3]. In their discussion of erythema multiforme, Trayes et al. emphasized that hypersensitivity-mediated target lesions can occur in response to infections or drugs, which may confound recognition of underlying etiologies when similar morphologies are produced by infectious organisms themselves [5]. In another vein, Kaszycki et al. reported early secondary syphilis with prominent granulomatous inflammation and sparing of the palms and soles, challenging the conventional association of granulomatous pathology with tertiary syphilis and suggesting that dermal immune responses to *T. pallidum* can vary widely even within the secondary stage [1].

Systemic manifestations in these reports further emphasize syphilis as a mimicker of vasculitic and autoimmune diseases. Sangesland and colleagues described a man presenting with acute unilateral vision loss, constitutional symptoms, elevated inflammatory markers, a maculopapular rash, and temporal artery tenderness, all of which suggested giant cell arteritis. High-dose corticosteroids were initiated, but subsequent testing revealed neurosyphilis, and appropriate antibiotic therapy led to near-normalization of vision [8]. This case echoes the diagnostic pathway that might have occurred in our patient had syphilis not been considered early: a vasculitic-appearing rash and systemic symptoms could easily lead to aggressive immunosuppression without addressing the underlying infection.

The pathophysiology of syphilis-associated vasculitis is not fully defined, but infectious triggers are well recognized in leukocytoclastic vasculitis more broadly. Mohamed et al. showed a plasma cell-rich perivascular infiltrate and spirochetes within the skin, suggesting that both direct endothelial infection and immune complex deposition may contribute to vascular damage in secondary syphilis [6]. Rodriguez et al. observed medium- to large-vessel fibrinoid necrosis with neutrophilic infiltrates in their rupioid syphilis case, again pointing to a robust immune-mediated vasculitic response to spirochetal antigens [7].

Therapeutically, the cornerstone of management in all these cases is the timely administration of penicillin. In the case described by Basheer et al., a single intramuscular dose of benzathine penicillin G led to rapid resolution of the rash and a marked decline in non-treponemal titers over subsequent weeks [4]. Puttur et al. similarly reported brisk improvement of painful targetoid lesions after benzathine penicillin G in an HIV-infected patient, alongside initiation of antiretroviral therapy [3]. Mohamed et al. and Rodriguez and Dhaliwal also documented significant clinical improvement with penicillin treatment in patients whose presentations were dominated by vasculitic or rupioid lesions [6, 7]. Consistent with these reports, our patient experienced rapid fading of purpura and resolution of pain following cephalosporin administration, with no need for systemic corticosteroids once syphilis was identified as the underlying cause.

From an epidemiologic standpoint, the importance of recognizing secondary syphilis in this context is magnified by its ongoing resurgence. Kaszycki et al. noted that syphilis cases in the United States have reached their highest incidence since 1950, underscoring a broad population-level vulnerability to missed diagnoses [1]. Basheer et al. highlighted that rising rates among heterosexual men and women, not only men who have sex with men, mean that clinicians cannot rely on narrow risk profiles when considering syphilis in the differential diagnosis of rash [4]. Sudibyo and Qurrohman’s report of secondary syphilis in a young woman with diffuse mucocutaneous involvement further reinforces that syphilis must remain on the radar across age groups and sexes [2].

Our case carries several practical implications. First, syphilis serology should be included early in the evaluation of cutaneous small-vessel vasculitis of unclear etiology, particularly in adults with systemic symptoms or potential sexual exposure. Second, while corticosteroids may be indicated in life-threatening vasculitic syndromes, their empiric use should ideally follow or coincide with a careful search for infectious triggers, including syphilis, to avoid immune suppression in the presence of an untreated, curable infection.

As a single case report, our observations cannot define the true frequency of vasculitic presentations in secondary syphilis, nor can they fully elucidate the immunopathogenesis of this association. Nonetheless, when considered alongside reports of leukocytoclastic vasculitis, rupioid lesions with vasculitis, granulomatous secondary syphilis, and neurosyphilis mimicking giant cell arteritis [1, 6-8]. Our case strengthens the message that syphilis should be actively sought and not merely considered in patients with vasculitic-appearing rashes.

## Conclusions

This case of a woman with a purpuric, annular rash first suspected to be a small-vessel vasculitis ultimately attributable to secondary syphilis highlights the enduring role of syphilis as a “great imitator” of both dermatologic and systemic disease. Clinically, the eruption mimicked cutaneous leukocytoclastic vasculitis, yet the underlying driver was a common, treatable sexually transmitted infection.

This case illustrates how secondary syphilis can present with a vasculitic-appearing rash that is clinically indistinguishable from primary small-vessel vasculitis. Incorporating a sexual history and routine syphilis serology into the workup of unexplained vasculitic eruptions can prevent misdiagnosis and avoid unnecessary immunosuppression. Early recognition of syphilis as an infectious trigger allows timely, curative penicillin therapy, improving patient outcomes and limiting further transmission.
